# Lissencephaly caused by a *de novo* mutation in tubulin *TUBA1A*: a case report and literature review

**DOI:** 10.3389/fped.2024.1367305

**Published:** 2024-05-14

**Authors:** Sijing Ren, Yu Kong, Ruihan Liu, Qiubo Li, Xuehua Shen, Qing-Xia Kong

**Affiliations:** ^1^ The Second Clinical Medical College, Shandong University of Traditional Chinese Medicine, Jinan, Shandong, China; ^2^ Department of Neurosciences, Affiliated Hospital of Jining Medical University, Jining, Shandong, China; ^3^ Department of Imaging, Affiliated Hospital of Jining Medical University, Jining, Shandong, China; ^4^ Department of Pediatrics, Affiliated Hospital of Jining Medical University, Jining, Shandong, China

**Keywords:** tubulinopathies, *TUBA1A* mutation, lissencephaly, epilepsy, neuronal migration

## Abstract

Tubulin plays an essential role in cortical development, and *TUBA1A* encodes a major neuronal *α*-tubulin. Neonatal mutations in *TUBA1A* are associated with severe brain malformations, and approximately 70% of patients with reported cases of *TUBA1A* mutations exhibit lissencephaly. We report the case of a 1-year-old boy with the *TUBA1A* nascent mutation c.1204C >T, p.Arg402Cys, resulting in lissencephaly, developmental delay, and seizures, with a brain MRI showing normal cortical formation in the bilateral frontal lobes, smooth temporo-parieto-occipital gyri and shallow sulcus. This case has not been described in any previous report; thus, the present case provides new insights into the broad disease phenotype and diagnosis associated with *TUBA1A* mutations. In addition, we have summarized the gene mutation sites, neuroradiological findings, and clinical details of cases previously described in the literature and discussed the differences that exist between individual cases of *TUBA1A* mutations through a longitudinal comparative analysis of similar cases. The complexity of the disease is revealed, and the importance of confirming the genetic diagnosis from the beginning of the disease is emphasized, which can effectively shorten the diagnostic delay and help clinicians provide genetic and therapeutic counseling.

## 1 Introduction

Tubulinopathy is a general term for rare diseases caused by tubulin gene mutations, which are usually characterized by complex brain malformations and severe neurological manifestations (1–3). *TUBA1A* is the most commonly mutated gene in patients affected by tubulinopathies (1) and is located on chromosome 12q13.12. The gene product can be divided into three functional, structural domains: the amino-terminal domain containing the nucleotide-binding region, the middle domain containing the paclitaxel-binding site, and the carboxyl-terminal domain that may constitute the binding surface of the motor protein (4, 5). The reported patient TUBA1A mutation site (p.Arg402Cys) is in the C-terminal domain (residues 382–451), which may constitute the binding surface of microtubule-associated proteins and molecular motors such as kinesin and dynein.

Microtubules are cytoskeletal fibers dynamically assembled from heterodimers of evolutionarily conserved α- and β-tubulin subunits. Microtubules are present in all eukaryotic cells and play important roles in brain development, including mitosis, neuronal proliferation, migration, and differentiation, such as synapse formation, connection, and axonal transport (2). Tubulin is a structural subunit protein that forms microtubules, and its highly dynamic cytoskeletal structure participates in a variety of cellular functions (6–8) and plays a key role in cortical development and stratification (9). Since it was first described in 2007 (1), at least eight gene variants have been reported clinically, including α-tubulin (*TUBA1A*, *TUBA8*), β-tubulin (*TUBB2A*, *TUBB2B*, *TUBB3*, *TUBB4A*, *TUBB*), and γ-tubulin (*TUBG1*) (10, 11), Among them, *TUBA1A* encodes a major neuronal α-tubulin that is highly expressed in the developing nervous system, and its *de novo* mutation disrupts brain development processes and is associated with severe brain malformations (12). These include lissencephaly, cerebellar hypoplasia, corpus callosum hypoplasia, and brainstem abnormalities (13, 14). This gene is often associated with dyskinesia, epilepsy, cognitive deficits, and other abnormalities (15, 16). To date, 77 missense variants have been reported, with most cases exhibiting *de novo* autosomal dominant inheritance (17).

Neuronal migration follows a three-step process (1): cell extension to a leading process (18); (2) nuclear migration to the leading process (“nucleokinesis”); and (3) retraction of the trailing process (19). Thus, nucleokinesis or nuclear migration is a key process for cell migration. Neuronal migration disorder is an inherited disorder of the central nervous system in which neurons originating from the cortical ventricles and subventricular zones move to their final location along radially oriented glial fibers perpendicular to the brain surface (20). Neuronal migration disorders are the result of many chromosomal deletions and gene mutations and can clinically manifest as severe neurodevelopmental disorders and epilepsy in childhood. Dynein is dependent on several factors for intracellular transport. Dynein adopts an autoinhibited conformational state (called “phi” particles) that reduces its microtubule on-rate and restricts its ability to interact with dynein and adaptors (21). After adopting the “open” (no inhibition) state, dynein easily assembles into a motility-competent dynein–dynactin–adaptor (DDA) complex (22). The factors that promote DDA assembly enhance dynein activity, whereas those that prevent it have the opposite effect. Therefore, the factors that affect microtubule function via dynein may be the key effectors inhibiting the “open” conformation, thereby inhibiting DDA assembly and motility. In summary, dynein is a critical effector of nuclear migration and is closely associated with neuronal migration. Both processes are critical during brain development and other physiological events.

Here, we report a case of *TUBA1A de novo* mutation c.1204C >T, p.Arg402Cys, resulting in lissencephaly, developmental delay, and seizures, with a brain MRI showing normal cortical formation in the bilateral frontal lobes, smooth temporo–parieto–occipital gyri, and shallow sulci. To our knowledge, this case has not been described in any previous report.

## 2 Case presentation

### 2.1 Case introduction

The patient was a 1-year-old infant. At the age of 5 months, he was admitted to a hospital because he could not raise his head and had no obvious cause of intermittent convulsions. The convulsions manifested as nodding (infantile) spasms, string attacks, and frequency. Sometimes, his limbs trembled, he was easily frightened, his upper limbs were held in a ball, and he shed tears. This symptom occurred 15–20 times a day. At the age of 7 months, there was initially no nodding spasm. Occasionally, there were stiff movements of both upper limbs, 90° between the hands and the body, scissor-like movements, which were instantly relieved, sometimes in a cluster, 3–5 times at once, lasting for 20 s and then relieved. After relief, his body was arched, which was obvious after waking up. Some antiepileptic drugs (including clobazam) were contraindicated, he was currently taking sodium valproate and vigabatrin, and had recent dry stools, oliguria, and anorexia, accompanied by sleep cycle disorders. The child was born by full-term cesarean section with a birth weight of 4.1 kg. His parents were healthy, and there were no obvious abnormalities in his family history. His mother said that she was healthy during pregnancy and had typical prepregnancy examination results. Perinatal asphyxia did not occur during the perinatal period, his Apgar score was unknown, and there were no neonatal problems. The child was exclusively breastfed, and his sucking ability was as expected, but his growth and development were slower than expected for his age.

### 2.2 Physical examination

Physical examination found a vacant expression, poor mental response, slightly concave occiput, poor vision, no recognition, closed anterior fontanel, equal size and roundness of the pupils bilaterally, sensitive light reflex, and soft neck. We found unstable muscle tension of the limbs, heavy dorsiflexion of the head, backward tilt of the neck, instability of the head over 90° in the prone position, and instability of the elbow support, and that the patient was negative for Kirschner's, Bourdon's, and Barthelson's signs.

### 2.3 Imaging

**Figure 1:**
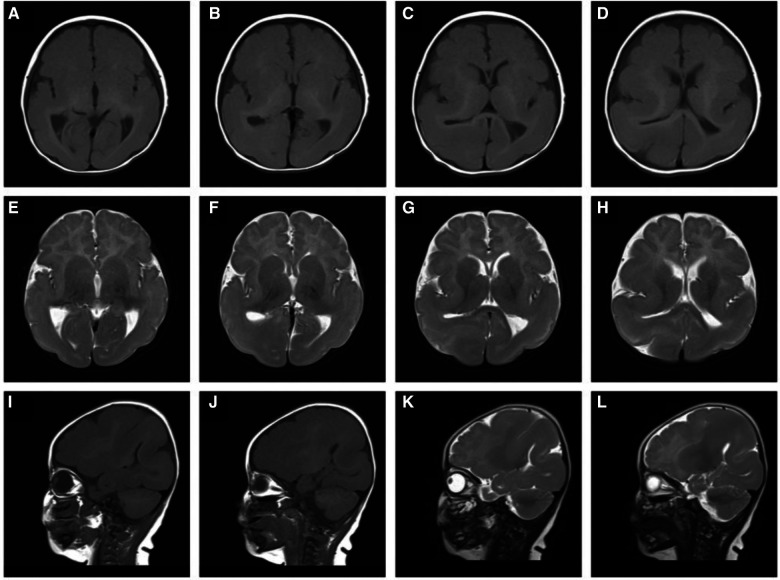
T1-weighted sagittal (**A**–**D**), T2-weighted sagittal (**E**–**H**), T1-weighted axial (**I**, **J**), T2-weighted axial (**K**, **L**) magnetic resonance imaging: showing abnormal changes bilaterally in the temporo–parietal occipital gyri and sulci, pachygyria and lissencephaly.

At the age of 5 months, brain MRI and diffusion-weighted imaging (Figure 1) showed normal cortex formation in the bilateral frontal lobes, wide temporal, parietal and occipital gyri, and shallow sulci. The imaging was interpreted as showing abnormal changes in the bilateral temporo–parietal occipital gyri and sulci, and pachygyria and agyria were considered.

### 2.4 Video-electroencephalogram

At the age of 5 months, the results of 6-h video electroencephalography (EEG) revealed severe abnormalities (Supplementary Figure S1): background activity was abnormal, and most of the waking periods were highly arrhythmic. Against a background of diffuse 2–7 Hz irregular slow waves, there were a large number of multifocal sharp waves, spike waves, slow waves, spike slow waves, sharp slow waves, multispike waves, multispike slow waves and fast wave rhythms, and the posterior head was obviously asymmetric and asynchronous. During the paroxysmal periods, two clinical episodes were monitored, manifesting as nodding spasms and holding his upper limbs in a ball in the vertical and horizontal positions. When lying flat, his upper limbs were abducted, and his lower limbs were lifted, in a cluster of 15–25 times. Simultaneously, the EEG showed extensive high-amplitude 1.5–2.5 Hz sharp slow wave bursts one or two times, followed by 14–20 Hz low-amplitude fast wave rhythm fast wave bursts for 2–3 s and low-amplitude electromyographic bursts. The background rhythm was then restored, supporting a diagnosis of infantile spasms (nodding) at the age of 7 months. The results of 6-h video EEG at the age of 11 months (Supplementary Figure S2) showed that the background was slow and that there were many focal discharges in each sleep stage, which were asynchronous high-amplitude sharp waves and sharp slow waves in the left and right parietal areas, the left and right central areas, and the right frontal area. During sleep, continuous slow waves of approximately 2 Hz were observed in the right or anterior cephalic leads, which were located in the right central area. Blinking, opisthotonos, right-hand elevation, and elevation of both hands were observed during the attack, which supported a diagnosis of epileptic encephalopathy.

### 2.5 Laboratory examinations

No results were outside the reference ranges for routine laboratory tests.

### 2.6 Genetic screening

**Figure 2:**
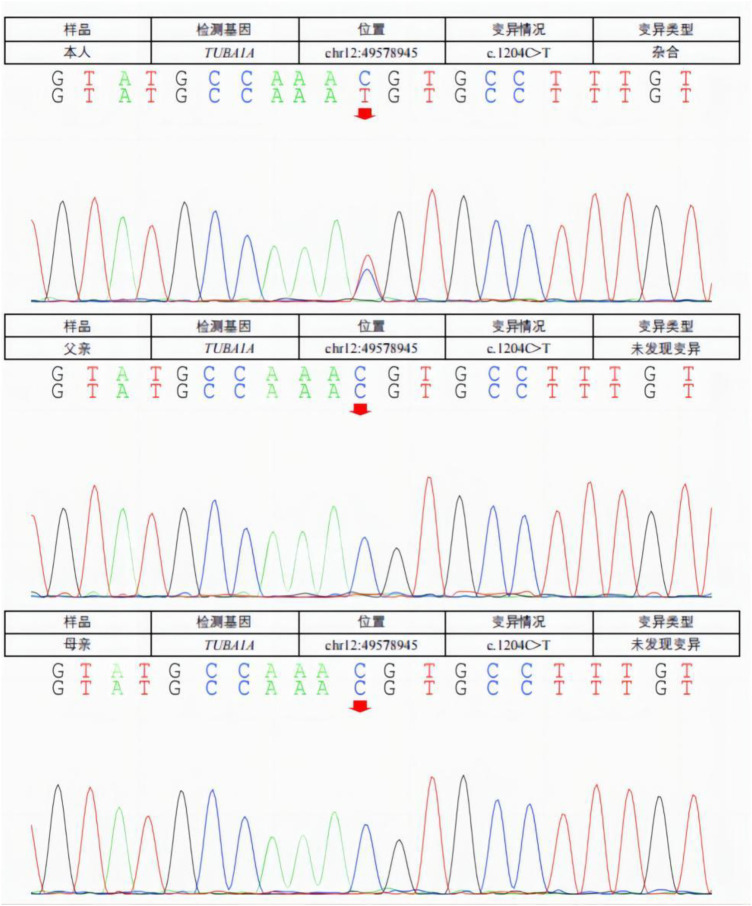
A missense mutation in the *TUBA1A* gene was detected in the patient. C. 1204C >T (C to T at nucleotide 1,204 of the coding region) is a heterozygous nucleotide variation resulting in p.Arg402Cys (Ary to Cys at amino acid 402). No such mutation (arrow) was found in the *TUBA1A* gene of his parents.

When the patient was 5 months old, 2 ml of peripheral venous blood was drawn from the patient and his parents, and genomic DNA was extracted from the blood using the standard method of whole exome sequencing. The test results (Figure 2) showed that the patient had a missense mutation in *TUBA1A*, c.1204C >T (nucleotide 1,204 in the coding region changed from C to T), which is a heterozygous nucleotide variation. This variation resulted in p.Arg402Cys (amino acid 402 changed from Arg to Cys). No such mutation was found in the TUBA1A gene of his parents. The patient was diagnosed with a *de novo TUBA1A* mutation and lissencephaly.

## 3 Discussion

Lissencephaly is a rare inherited neurological disorder caused by abnormal neuronal migration, resulting in the absence or loss of sulci and gyri, accompanied by abnormal histological structure of the cerebral cortex (23). Affected children present with microcephaly, global developmental delay, early onset of epileptic infantile spasms in infancy, and intellectual disability (24). Approximately 70% of patients with reported cases of *TUBA1A* mutations exhibit lissencephaly (13); here, we report a unique case confirming that the *TUBA1A de novo* mutation causes lissencephaly.

**Figure 3:**
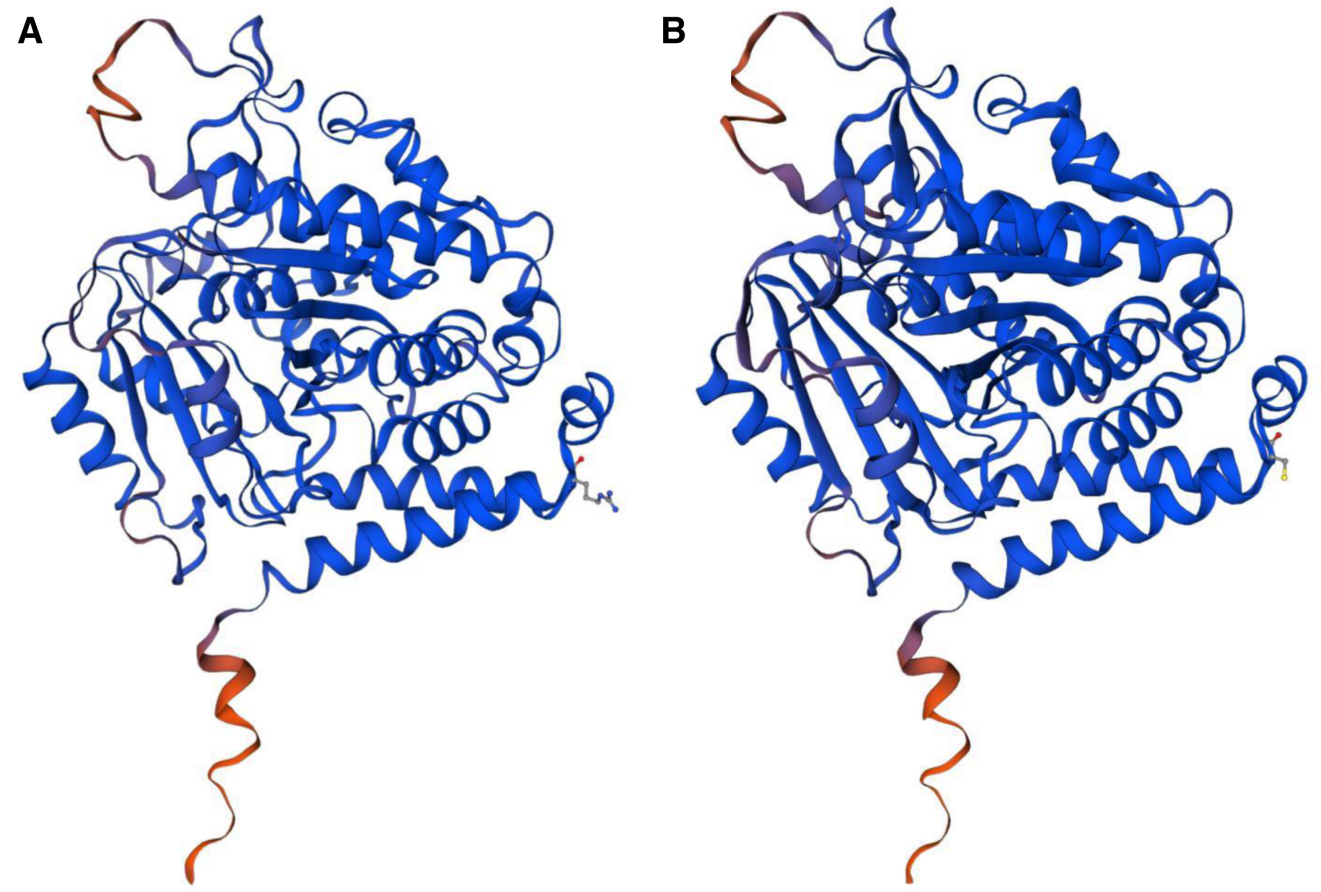
(**A**) The normal protein tertiary structure of the *TUBA1A* gene. (**B**) The protein tertiary structure of the *TUBA1A* mutant gene.

The most striking feature of this case is the *TUBA1A de novo* mutation c.1204C >T, p.Arg402Cys, which causes lissencephaly, developmental delay, and seizures. Because the human cerebral cortex accounts for approximately 82% of the total mass of the brain (25), the refined cortical architecture is accomplished by a complex and composite organization of neurons and nonneuronal cells from different lineages. Tubulin is involved in all three stages of cortical development: neuronal progenitor proliferation, neuronal migration, and neuronal differentiation. *TUBA1A* is transiently expressed during neuronal development (26) and is selectively encoded and predominantly expressed in α-1 tubulin in postmitotic neurons, and mutations in this gene cause changes in spatial structure, leading to alterations in protein translation and modification, as predicted by the three-dimensional view (Figure 3). *TUBA1A* missense mutations affect TUBA1A protein homeostasis, reduce the abundance of TUBA1A in cells, promote tubulin aggregation, prevent TUBA1A from entering microtubules, affect tubulin heterodimer formation, interfere with microtubule function, and lead to strong defects in neuronal migration. The function and activity of α-tubulin are reduced, eventually leading to neuropathy (27). Substitutions that affect the conserved R402 residue represent 30% of TUBA1A mutations identified in patients, suggesting that this site is essential for the role of TUBA1A in brain development. R402 mutant tubulins assemble into microtubules that support regular kinesin motor activity but fail to support the activity of dynein motors. Studies have shown that the missense mutations selectively impair dynein motor activity and severely and strongly disrupt cortical neuronal migration. Notably, the level of dynein impairment scales with the expression level of the mutant, suggesting a “poisoning” mechanism (5, 28). Cytoplasmic dynein is a motor protein whose movement on the microtubule (MT) track is achieved by an ATP hydrolysis-dependent or MT-binding-driving pathway mediating two-way communication of the coiled-coil stalk between the microtubule-binding domain and the ATPase domain. It is involved in many cellular processes (29, 30). These findings suggest that R402 mutants cause lissencephaly by poisoning dynein activity when its function is crucial for neurodevelopment. This suggestion strongly supports a causal role in the pathology of brain malformation. However, the mechanism by which *TUBA1A* causes pathological changes has not been reported clearly, and further studies on the structure, function, and related neurodevelopmental phenotypes of this gene are warranted for its clarification.

By summarizing the gene mutation sites, neuroradiological findings, and clinical details of all cases previously described in the literature (Supplementary Table S1), in 2007, Keays et al. first reported the identification of TUBA3 mutations (the human homolog of *TUBA1A*) in two patients who presented with type I anencephalic gyrus malformations, megalencephalic gyrus malformations, and hippocampal abnormalities (1). Poirier et al. reported a case of dysplasia of brain spectrum genes with the same mutation site in *TUBA1A*, c.1204C >T, p.Arg402Cys. Compared with the present case, their patient only showed normal cortex formation in bilateral frontal lobes, wide temporal, parietal and occipital gyri; and shallow sulci. The patient not only had cortical dysplasia, the mutation was also associated with an abnormal corpus callosum, hypoplasia of the inferior vermis of the cerebellum, and hypoplasia of the brainstem, characterized by severe classical lissencephaly and dilated ventricles (31). These features extend the MRI findings of the reported case, complement the clinical manifestations, and provide clues for further comparative study of the mutation site. These phenotypic differences emphasize that the best approach to determine the disease is to verify it through further genetic testing, and genetic methods are the key to making the final diagnosis.

Indeed, previous reports have shown that the TUBA1A mutant phenotype is associated with a broad spectrum of cortical and subcortical brain malformations. Major cortical abnormalities include “classical” lissencephaly, lissencephaly with agenesis or dysgenesis of the corpus callosum, lissencephaly with cerebellar hypoplasia, polymicrogyria, and mild-to-moderate dysgyria (32). The basal ganglia, thalamus, corpus callosum, and dysplasia of the cerebellar vermis and cerebellar cortex may also be involved. Brainstem malformations often present as asymmetric hypoplasia, and these findings can be clues for the diagnosis of *TUBA1A* mutations. The brain MRI findings in the case presented in this report revealed abnormal changes in the bilateral temporo–parieto–occipital gyri, sulci, and lissencephaly, consistent with the major *TUBA1A* mutation-associated phenotype. Oegema et al. used targeted sequencing to identify two cases of *TUBA1A* mutations that were *de novo* or inherited from chimeric parents. Affected patients showed severe developmental delays and seizures, which are highly consistent with the clinical manifestations of the reported case, except for the MRI findings of diffuse irregular convolution and fissure of the cortex, dysplasia of the cerebellar vermis, asymmetry of the pons, dysplasia of the medulla oblongata (1 case), partial hypoplasia of the corpus callosum, enlargement of the ventricles, and cranial nerve dysplasia (33). These differences emphasize the importance of neuropathological examination in patients with lissencephaly, which can provide clues for understanding the underlying *TUBA1A* mutation lesions to elucidate various pathogenesis and pathophysiological mechanisms.

Widely reported clinical manifestations of lissencephaly caused by *TUBA1A* mutations include cognitive impairment, developmental delay, and seizures, which were also present in the present case. Similarly, Sohal et al. reported the case of a 14-month-old girl with *TUBA1A* mutant lissencephaly whose brain MIR at the age of 4 months showed incomplete lissencephaly mainly affecting the parieto–occipital cortex and had cerebellar and brainstem hypoplasia, except for epileptic infantile spasms, lack of oropharyngeal coordination, and wheezing (24). Hikita et al. reported that an 8-year-old girl with a *de novo* mutation in *TUBA1A* presented with lissencephaly, microcephaly, and early-onset seizures. Hirschsprung disease and the syndrome associated with inappropriate antidiuretic hormone have also been identified (34). These conditions have not been previously described in *TUBA1A* mutation-associated diseases, providing new insights into the phenotype of these widespread diseases. In addition, microcephaly, intellectual disability, varying degrees of facial deformities, quadriplegia, and other related lesions, which were not present in the reported cases, may also support the diagnosis of lissencephaly due to suspected *TUBA1A* mutations.

It is important to note that in the present case, there were pronounced seizures in early childhood, manifested as infantile spasms and spastic encephalopathy. According to the literature, TUBA1A tubulinopathy is a relevant cause of congenital brain malformations as well as early-onset and intractable epilepsy with semiologic diversity (15). Epilepsy is common in patients with *TUBA1A* lissencephaly who develop complex epilepsy in late childhood, including atypical absence seizures, myoclonic and atonic seizures, partial complex seizures, and tonic–clonic seizures (35), although this requires further follow-up verification. Epilepsy was reported in 28% (44/155) of patients with *TUBA1A* mutations described in the literature, with seizures ranging from birth to 3 years old. The most common seizure types were focal (39% *TUBA1A*) and spastic (26% *TUBA1A*) (36). Severe symptomatic epilepsy syndrome is observed in 70%–80% of patients with brain malformations (37), and seizures are controlled in 33% of patients carrying *TUBA1A* gene mutations. The most frequently used drugs were sodium valproate and phenobarbital. However, in the most severe cases, the prognosis is still poor, and further studies are still needed to elucidate the mechanism of disease development caused by gene mutations, find effective therapeutic targets, effectively shorten the delay in diagnosis and treatment, and improve the prognosis of the disease.

## 4 Conclusion

The brain MRI findings and clinical phenotype in the present patient are consistent with the major *TUBA1A* mutation-associated phenotype, and based on previous findings that the mutation in *TUBA1A* determines the brain disease phenotype, the patient was diagnosed with lissencephaly caused by a mutation in *TUBA1A*. Here, we describe a new case with a striking feature of *TUBA1A de novo* mutation c.1204C >T, p.Arg402Cys, resulting in lissencephaly, developmental delay, and seizures, and a brain MRI showing normal cortical formation in the bilateral frontal lobes, smooth temporo–parieto–occipital gyri, and shallow sulci. The summary and description of similar cases emphasize the importance of confirming a genetic diagnosis from the beginning of the disease. Accumulating cases of *TUBA1A* mutation related to the disease and clarifying the mutation-specific spectrum of its neurodevelopmental and clinical phenotypes are warranted. Information on disease-associated *TUBA1A* mutations should be evaluated through detailed case studies to establish appropriate genetic counseling. To our knowledge, there are no reports on the pathogenesis and effective treatment of lissencephaly caused by *TUBA1A* mutation, so future research is warranted to determine the pathogenesis and effective treatment of *TUBA1A* mutation.

## Data Availability

The original contributions presented in the study are included in the article/**Supplementary Material**, further inquiries can be directed to the corresponding authors.
