# Supplementary material for: Lissencephaly caused by a de novo mutation in tubulin TUBA1A: a case report and literature review
Source: Front Pediatr. 2024 May 14;12:1367305. doi: 10.3389/fped.2024.1367305 (PMC11135126; doi:10.3389/fped.2024.1367305)
Supplement: Supplementary file 1 [file Datasheet1.pdf]

Supplementary Material

1 Supplementary Table

Supplementary Table 1. Gene mutation site, brain MRI and clinical phenotype of patients with *TUBA1A* mutation

Author/Ref.	*TUBA1A* case	Gene mutation site	Brain MRI	Clinical phenotype
Poirier et al. (23)	3	c.790C>T,p.Ary264Cys<br>c.1205G>A,p.Ary402His<br>c.1204C>T,p.Ary402Cys	Cortical hypoplasia<br>Abnormality of cerebellum, hippocampus and corpus callosum<br>Brain stem abnormality	lissencephaly<br>Stunting
Fallet-Bianco et al. (29)	4 Fetus	Not recorded	Cortical and hippocampal abnormalities<br>Cortical and cerebellar ectopic neurons<br>Brainstem ectopic neuron	None
Bahi-Buisson et al. (30)	6	c.1190T>C,p.Leu397Pro<br>c.1265G>A,p.Ary422His<br>c.1264C>T,p.Ary422Cys<br>c.1306G>T,p.Gly436Ary<br>c.790C>T,p.Ary264Cys	Perisylvian pachygyrus<br>Cerebellar hypoplasia	Congenital Microcephaly<br>intellectual disability<br>Diplegia/quadriplegia
Morris-Rosendahl et al. (31)	5	Not recorded	Agenesis of corpus callosum<br>Cerebellar hypoplasia<br>Multiple cortical malformations	Congenital Microcephaly
Jansen et al. (32)	3	c.629A>G,p.Tyr210Cys<br>c.13A>C,p.Ile5Leu	Thin corpus callosum<br>Hypoplasia of inferior vermis<br>Hypoplasia of the middle and lower cerebellar hemispheres	1 case of lissencephaly<br>Polygyria in 2 cases<br>Spastic diplegia<br>Ataxia<br>intellectual disability<br>Epileptic
Sohal et al. (18)	1	c.1205G>T,p.Ary402Leu	Agenesis of corpus callosum<br>Ventriculomegaly<br>Cerebellar brainstem hypoplasia	Microcephaly<br>Facial deformity<br>Tonic spasm<br>Epileptic infantile spasms<br>Oropharyngeal incoordination and stridor
Okumura et al. (33)	1	c.1096G>A,p.Gly366Ary	Ventricular dilatation with thin cortex<br>Basal ganglia poorly differentiated<br>Agenesis of corpus callosum<br>Cerebellar hypoplasia with preserved vermis	lissencephaly<br>Stunting<br>Facial deformity
Hikita et al.	1	c.599G>A,p.Cys402Tyr	Agyria	lissencephaly

(25)			Enlarged lateral ventricles<br>Agenesis of corpus callosum<br>Abnormal hippocampal rotation<br>Malformation of basal ganglia and thalamus<br>Cerebellar dysplasia	Microcephaly<br>Early onset seizures<br>Hirschsprung disease<br>Inappropriate antidiuretic hormone secretion
Shimojima et al. (34)	1	c.79G>C,p.Glu27Gln	Dilatation of cephalic and lateral ventricles<br>Simplified gyral pattern<br>Agenesis of corpus callosum and vermis of cerebellum	Microcephaly<br>Severe developmental delay
Bosemani et al. (35)	1		Helminth hypoplasia<br>Cephalocaudal medulla<br>Abnormal increase in midbrain<br>Absence of dorsal plane of brainstem	Stunting
Oegema et al. (24)	2	p.Arg214His	Cerebellar vermis dysplasia<br>Pontine asymmetry<br>Medullary dysplasia<br>Partial agenesis of corpus callosum<br>Basal ganglia dysplasia<br>Ventriculomegaly<br>Cranial nerve dysplasia	Microcephaly<br>Severe developmental delay<br>Epileptic
Mencarelli et al. (36)	1	c.320A>G,p.His107Arg	Mild asymmetry and dilation of the lateral ventricles<br>lissencephaly with peripheral thick gyri and diffuse posterior thick gyri<br>Internal capsule dysplasia<br>Cerebellar dysplasia<br>Hypertrophy of corpus callosum	Seizures<br>Stunting<br>Hypotonia<br>Microcephaly<br>Mild facial deformity
Romaniell et al. (37)	5	p.Arg390His<br>p.Arg2Ser<br>p.Arg123His<br>p.Ser54Asn<br>p.Gly59Ser	Cerebellar cortical dysplasia	Stunting<br>Intellectual disability<br>Congenital microcephaly<br>Epileptic

2 Supplementary Figures

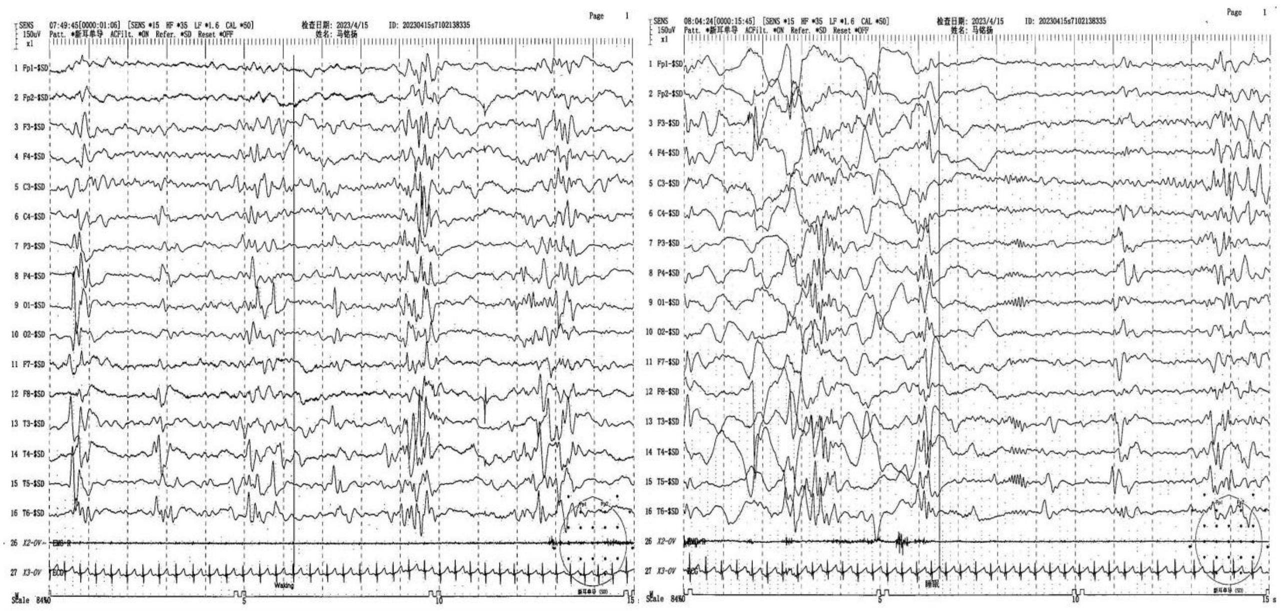

Supplementary Figure 1. Electroencephalogram (EEG) of the patient at 5 months: Most of the patients were highly arrhythmic in each stage of waking up. On the basis of diffuse 2-7 Hz irregular slow waves, there were a large number of multi-focal sharp waves, spike waves, slow waves, spike slow waves, sharp slow waves, multi-spike waves, multi-spike slow waves and fast wave rhythms, and the posterior head was obviously asymmetric and asynchronous.

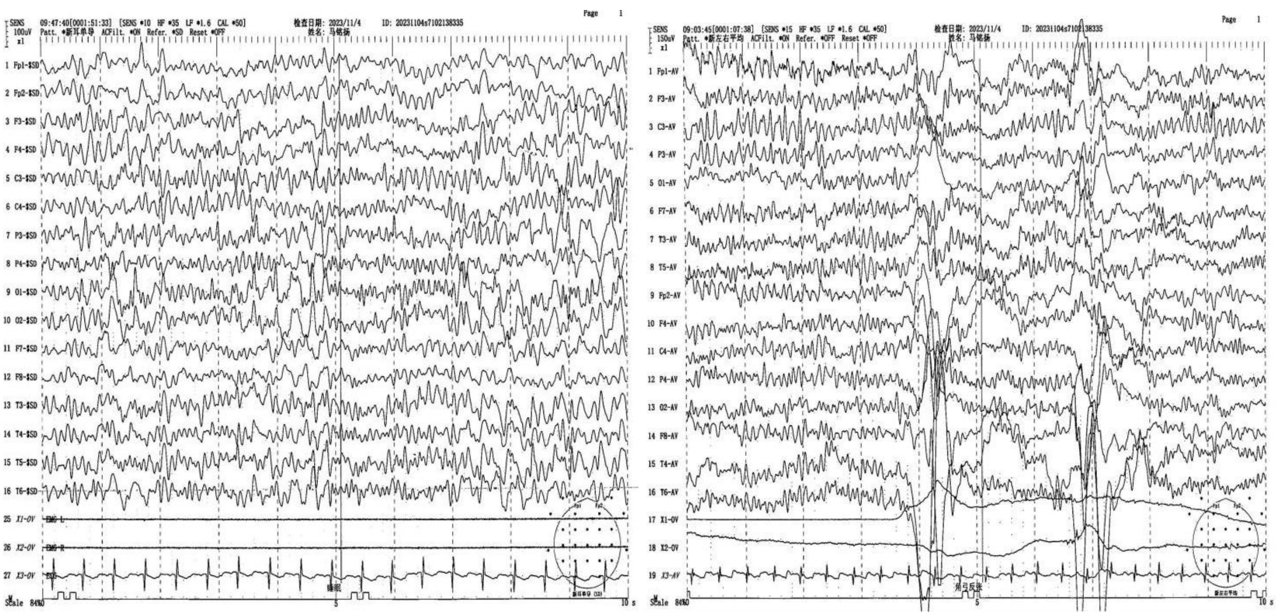

Supplementary Figure 2. Electroencephalogram of the patient at the age of 11 months: a few focal discharges were seen in each stage of waking up, which were asynchronous medium and high amplitude, sharp waves and sharp slow waves in the left and right parietal areas, the left and right central areas, and the right frontal area. During sleep, continuous slow waves of about 2 Hz were seen in the right or anterior cephalic leads, which were located in the right central area.
